# Penicillin production in industrial strain *Penicillium chrysogenum* P2niaD18 is not dependent on the copy number of biosynthesis genes

**DOI:** 10.1186/s12896-017-0335-8

**Published:** 2017-02-16

**Authors:** Sandra Ziemons, Katerina Koutsantas, Kordula Becker, Tim Dahlmann, Ulrich Kück

**Affiliations:** Lehrstuhl für Allgemeine und Molekulare Botanik, Ruhr-Universität Bochum, ND7/131, Universitätsstraße 150, 44780 Bochum, Germany

**Keywords:** *Penicillium chrysogenum*, Beta-lactam antibiotics, Production strain, Gene cluster amplification

## Abstract

**Background:**

Multi-copy gene integration into microbial genomes is a conventional tool for obtaining improved gene expression. For *Penicillium chrysogenum*, the fungal producer of the beta-lactam antibiotic penicillin, many production strains carry multiple copies of the penicillin biosynthesis gene cluster. This discovery led to the generally accepted view that high penicillin titers are the result of multiple copies of penicillin genes. Here we investigated strain P2niaD18, a production line that carries only two copies of the penicillin gene cluster.

**Results:**

We performed pulsed-field gel electrophoresis (PFGE), quantitative qRT-PCR, and penicillin bioassays to investigate production, deletion and overexpression strains generated in the *P. chrysogenum* P2niaD18 background, in order to determine the copy number of the penicillin biosynthesis gene cluster, and study the expression of one penicillin biosynthesis gene, and the penicillin titer. Analysis of production and recombinant strain showed that the enhanced penicillin titer did not depend on the copy number of the penicillin gene cluster. Our assumption was strengthened by results with a penicillin null strain lacking *pcbC* encoding isopenicillin N synthase. Reintroduction of one or two copies of the cluster into the *pcbC* deletion strain restored transcriptional high expression of the *pcbC* gene, but recombinant strains showed no significantly different penicillin titer compared to parental strains.

**Conclusions:**

Here we present a molecular genetic analysis of production and recombinant strains in the P2niaD18 background carrying different copy numbers of the penicillin biosynthesis gene cluster. Our analysis shows that the enhanced penicillin titer does not strictly depend on the copy number of the cluster. Based on these overall findings, we hypothesize that instead, complex regulatory mechanisms are prominently implicated in increased penicillin biosynthesis in production strains.

**Electronic supplementary material:**

The online version of this article (doi:10.1186/s12896-017-0335-8) contains supplementary material, which is available to authorized users.

## Background

Fungi can produce diverse secondary metabolites with antibacterial activity against numerous microorganisms. Among these metabolites, penicillin represents the starting point of the discovery of highly effective antibiotics, a milestone in therapeutic medicine [1, 2]. To date, only the filamentous ascomycete *Penicillium chrysogenum* is used industrially to obtain economically relevant penicillin titers [3].

In the first reaction of penicillin biosynthesis, the three precursor amino acids L-α-aminoadipic acid, L-cysteine, and L-valine are condensed to the tripeptide δ-(L-α-aminoadipyl)-L-cysteinyl-D-valine (ACV). This step is catalyzed by ACV synthetase, a single multifunctional enzyme with non-ribosomal peptide synthetase activity that is coded by the *pcbAB* gene (synonym, *acvA*). The second step is characterized by the oxidative ring closure of the linear ACV tripeptide, leading to the formation of a bicyclic ring comprising the β-lactam and thiazolidine ring. This reaction is catalyzed by the isopenicillin N synthase, encoded by the *pcbC* gene (synonym, *ipnA*). The resulting compound, isopenicillin N, is the first bioactive intermediate of the penicillin biosynthesis pathway. In the third reaction of penicillin biosynthesis, the hydrophilic L-α-aminoadipate side chain of isopenicillin N is exchanged for a hydrophobic phenylacetyl or phenoxyacetyl group, resulting in the formation of penicillin G and penicillin V, respectively. This final step is catalyzed by the acyl-coenzyme A: isopenicillin N acyltransferase, and the corresponding gene is *penDE* (synonym, *aatA*) (for an overview see [2, 4, 5]).

All three penicillin biosynthesis genes occur in a single cluster that is structurally conserved in pro- and eukaryotic microbial producers. This shared characteristic supports the hypothesis that fungi have acquired these genes from bacteria through horizontal gene transfer [6, 7].

The progenitor of all industrially used *P. chrysogenum* strains is strain NRRL 1951 (= CBS 307.48), which was isolated in 1943 from a moldy cantaloupe in Peoria, IL. Since then, this strain and its descendants have been subjected to strong mutagenic treatments during strain improvement programs. This pressure has not only resulted in sharply increased antibiotic production but also in increased copy number of the penicillin biosynthesis cluster in some high-production strains, several of which harbor as many as 50 copies of the cluster [3, 8–10]. For example, in the high producer AS-P-78, a 106.5-kb DNA region comprising the pen cluster is amplified in tandem repeats of five or six copies linked by conserved hexanucleotide sequences, whereas wild-type strains contain a single copy of this region [11]. Fierro et al. [11] proposed that the amplification occurred by mutation-induced site-specific recombination at the conserved hexanucleotide sequences. The amplified region is not identical in the different high-producing strains tested, although the mechanism of amplification is probably similar.

Another descendant of strain NRRL 1951, obtained by X-ray and UV mutagenesis, is the former industrial strain *P. chrysogenum* P2 (ATCC 48271) [12], which shows a 85-fold increased penicillin titer compared to its ancestor. This strain was used for conventional mutagenesis to construct P2niaD18, a nitrate reductase-deficient derivative [13]. Recently, whole genome sequencing of this strain revealed that chromosome I carries a tandem repeat duplication of the penicillin biosynthesis cluster comprising genes *pcbAB*, *pcbC*, and *penDE* [14].

Here, we performed pulsed-field gel electrophoresis (PFGE) to further determine the size of the duplicated region. The PFGE revealed that a genomic region of about 110 kb, which harbors the pen cluster, is duplicated in the high-producer strain compared to the wild-type strain. Most strikingly, the loss of one of these copies did not result in decreased penicillin production, thus indicating that the copy number is not responsible for high production in P2niaD18. Although the penicillin biosynthesis pathway is well-studied and the enzymes involved are characterized in detail [2, 4], little is known about the complex regulatory mechanisms behind this process. Our results indicate that instead, regulation of penicillin biosynthesis may have a far more important effect on the amount of penicillin that the fungus produces and therefore represents an important starting point for targeted strain improvement programs.

## Methods

### Strains and culture conditions

All *P. chrysogenum* strains used in this study are listed in Table 1. Strain P2niaD18 [13], whose genome was recently determined by high-throughput sequencing [14], served as the fungal recipient for all experiments. Like all commonly used industrial strains, P2niaD18 is a derivate of the former industrial strain Q176, which have underdone multiple rounds of conventional mutagenesis [15]. Based on this strain, a marker-free deletion strain of gene *Pcku70* (∆Pcku70) was generated [16]. ∆Pcku70 served as a recipient for the construction of knockout mutants based on the FLP/*FRT* recombination system. All *P. chrysogenum* strains were grown in liquid complex medium or minimal medium at 27 °C and 120 rpm or grown on solid medium as already described [17]. To inoculate shake flasks and solid medium, we used spores collected from 7-day-old cultures grown on medium M322. Transformation of individual *P. chrysogenum* strains was performed as described previously [13, 18], and selection of transformants was done by growth on solid medium supplemented with 200 μg ml^−1^ nourseothricin, 40 μg ml^−1^ phleomycin, or 700 μg ml^−1^ pyrithiamine.

**Table 1:** *P. chrysogenum* strains used in this study

Strains	Relevant genotypes	Source
NRRL 1951 (= CBS 307.48)	Wild type, isolated from moldy cantaloupe; parent of most high yielding penicillin producing strains	[50]
P2niaD18	Penicillin producer; *niaD* ^*−*^	[13]
ΔpcbC T7-1	∆*Pcku70::FRT;* ∆*pcbC::FRT::*P*trpC::phleo::FRT; niaD* ^*−*^	This study
ΔPcku70 EK2	∆*Pcku70*::P*trpC*::*nat1*	[31]
ΔPcku70.1 T1	*∆Pcku70*::*FRT*::P*trpC*::*ble*::*FRT*	[16]
ΔPcku70.1 T17	*∆Pcku70*::*FRT*::P*trpC*::*ble*::*FRT*	[16]
ΔPcku70.2 T1-1	P*trpC*::*Pcflp*; P*ptrA*::*ptrA*; ∆*Pcku70*::*FRT*	[16]
ΔPcku70.2 T17-1	P*trpC*::*Pcflp*; P*ptrA*::*ptrA*; ∆*Pcku70*::*FRT*	[16]
ΔPcku70.2 T1-2	∆*Pcku70::FRT; niaD* ^*−*^	[16]
ΔPcku70.2 T17-2	∆*Pcku70::FRT; niaD* ^*−*^	[16]
ΔpcbC::pcbC	∆*Pcku70::FRT;* ∆*pcbC:: pcbC::FRT::* P*trpC::nat1::FRT; niaD* ^*−*^	This study
ΔpcbC::pcbC-gfp	∆*Pcku70::FRT;* ∆*pcbC::FRT::*P*trpC::phleo::FRT;* P*gpd::pcbC::egfp::*T*trpC;* P*trpC::nat1; niaD* ^*−*^	This study
ΔpcbC::pPCPV1	∆*Pcku70::FRT;* ∆*pcbC::FRT::*P*trpC::phleo::FRT;* P*penDE::penDE::*T*penDE;* P*pcbC::pcbC::*T*pcbC::*T*pcbC;* P*pcbAB::pcbAB::*T*pcbAB; niaD* ^*+*^	This study

Recombinant plasmids were generated using either standard laboratory techniques [19] or the In-Fusion® HD Cloning Kit (Clontech) according to the manufacturer’s instructions, with *Escherichia coli* strain XL1-Blue MRF’ as host for general plasmid construction and maintenance [20].

### Construction of plasmids

All plasmids used in this study are listed in Table 2. For generation of a *pcbC* deletion plasmid, the 5’ and 3’ regions of *PcvelB* in plasmid pKOvelB (a derivative of pD-Phleo) were replaced by 1-kb 5’ and 3’ flanking regions of *pcbC*, via *Sph*I and *Mlu*I (5’ flank) and *Nhe*I and *Not*I restriction sites (3’ flank), respectively, resulting in plasmid pKOpcbCFRTble. In an alternative approach, the 5’ flank of *PcvosA* in plasmid pKOvosA [21] was replaced by *pcbC*-specific flanks, using the *Sfu*I and *Nde*I restriction sites. The 3’ *PcvosA* flank was replaced by the 3’ *pcbC* flank using the In-Fusion® HD Cloning Kit (Clontech) according to the manufacturer’s instructions, resulting in plasmid pKOpcbCFRTnat. For complementation by homologous integration, the *pcbC* open reading frame (ORF) was introduced behind the 5’ *pcbC* flank of plasmid pKOpcbCFRTnat by using the *Nde*I restriction site. To achieve complementation by ectopic integration of a *pcbC-egfp* fusion construct, the *pcbC* ORF was integrated using the *Nco*I and *Not*I sites of p1783-1nat. Finally, for complementation of the *pcbC* null mutant with the complete penicillin biosynthesis cluster, plasmid pPCPV1 was used. This plasmid has a size of 39.8 kb and also carries the bacterial ampicillin resistance gene and the *A. nidulans niiA* and *niaD* genes, a 24.5 kb fragment with the penicillin biosynthesis gene cluster (*pcbAB*, *pcbC*, *penDE*) from *P. chrysogenum* (Kamerewerd and Kück, unpublished)*.* The nucleotide sequence of the penicillin gene cluster is identical in NRRL1951 (Dahlmann, unpublished data), Wisconsin 1255-54 [22], and P2niaD18 [23], and the same result was reported for another high titer strain BW1901 [8].

**Table 2:** Plasmids used in this study

Plasmid	Characteristics	Source
pD-Phleo	*trpC(p)::ble*	[46]
pKOvelB	1 kb 5’ flank region and 1 kb 3’ flank region of *PcvelB* with *FRT* sites in pD-Phleo	This study
pKOpcbCFRTble	Replacement of *PcvelB*-specific flanks by *pcbC*-specific flanks in pKOvelB	This study
pKOvosA	5’*PcvosA* flank, 5’*FRT*, P*trpC*, *nat1* resistance gene, 3’*FRT* sequence, 3’*PcvosA* flank	[16]
pPTRII_PcFLP	*trpC* promoter, *Pcflp* gene, *ptrA* resistance gene of *A. oryzae*, *AMA1* sequences of *A. nidulans*	[16]
pKOpcbCFRTnat	Replacement of *PcvosA*-specific flanks by *pcbC*-specific flanks in pKOvosA	This study
ppcbCFRTnat	Introduction of *pcbC* ORF behind 5’ *pcbC* flank in pKOpcbCFRTnat	This study
pGFP-pcbC	P*gpd::pcbC::egfp::*T*trpc,* P*trpC::nat1*	This study
pPCPV1	Plasmid harbouring the complete penicillin biosynthesis cluster and the *niaD* gene	Kamerewerd and Kück, unpublished

### Construction of knockout mutants and complementation strains

To generate a *pcbC* null mutant, strain ∆Pcku70.2 was used as a recipient. After restriction of plasmid pKOpcbCFRTble with *Pvu*II, the knockout cassette harboring the 5’ and 3’ flanking regions of pcbC, two FRT sites, and a phleomycin resistance cassette was introduced into the genome of ∆Pcku70.2 by homologous recombination. The recombination event was verified by PCR and Southern analysis. Sequences of oligonucleotides used in these studies are given in Table 3.

**Table 3:** Oligonucleotides used in this study

Name	Sequence (5’➔3’)	Specificity
3’pcbC_s	TTCGTCGAGAACGGTGAAGC	downstream region of *pcbC* 3’flank used for homologous recombination
Phleo_s	TCCTGCGCCTGATACAGAAC	3’region phleomycin resistance cassette
PtrpC_a	TGTTGACCTCCACTAGCTCC	5’region P*trpC*
5’pcbC_a	TAGTGGCCGAGAAGCCTATC	upstream region of *pcbC* 5’ flank used for homologous recombination
pcbC_s	TATACCATGGATGGCTTCCACCCCCAAGG	*pcbC*
pcbC_a	TACTCCATGGTAGTCTGGCCGTTCTTGTTG	*pcbC*
SSU1	ATCCAAGGAAGGCAGCAGGC	SSUr RNA
SSU2	TGGAGCTGGAATTACCGCG	SSUr RNA
PcpcbC-RT_s	CCCTCCCGTTCTTCGTCAATC	*pcbC* (qRT-PCR)
PcpcbC-RT_a	CTGCAGATAGTAGCCGTACGA	*pcbC* (qRT-PCR)

For complementation by homologous recombination, the *Pvu*II fragment of ppcbCFRTnat, harboring the complementation cassette with the 5’ and 3’ flanking regions of *pcbC*, the *pcbC* ORF, and a nourseothricin resistance cassette, was introduced into the *pcbC* null mutant. For ectopic complementation of a *pcbC-egfp* fusion construct, ∆pcbC was transformed with plasmid pGFP-pcbC resulting in the construction of ∆pcbC::pcbC-gfp. Strains of both complementation variants were verified by PCR and Southern analysis.

For complementation of ∆pcbC with the complete penicillin biosynthesis cluster, plasmid pPCPV1 was transformed into the deletion mutant. The *niaD* gene on pPCPV1 served as a selection marker, thus complementing the nitrate reductase deficiency of the deletion mutant.

### Preparation and analysis of nucleic acids

Isolation of fungal genomic DNA was carried out as described previously [24, 25], and DNA was isolated from hyphal cells grown at 27 °C and at 120 rpm for 72 h in liquid media. Southern blotting was performed with a GeneScreen hybridization transfer membrane (PerkinElmer, USA), hybridized with [α-^32^P]dCTP-labeled probes using standard methods [19].

### Pulsed-field gel electrophoresis (PFGE)

Protoplasts were treated as described previously to isolate intact chromosomes [26]. The CHEF Mapper system (Bio-Rad, Richmond, CA) was used to separate large DNA fragments [27], which were obtained by hydrolysis with the rare-cutter endonucleases *Pac*I, *Pme*I, and *Swa*I, as specified in the results section. Pulse times were done for 18 h at 6 V/cm with initial switching intervals of 10 s and final switching intervals of 20 s.

### qRT-PCR for quantification of the *pcbC* transcript

RNA extraction for quantitative reverse transcriptase PCR (qRT-PCR) was performed using the RNeasy® Plus Universal Midi Kit (QIAGEN, Hilden, Germany) according to the instructions provided by the manufacturer. qRT-PCR analysis was performed as described previously [13, 28]. Amplification of the SSUrRNA (small subunit ribosomal RNA) was used as a reference for normalization. Sequences of oligonucleotides used for qRT-PCR are given in Table 3.

### Penicillin bioassay

For a penicillin bioassay, 100 ml of liquid complex medium were inoculated with 1 × 10^7^ spores. Cultures were incubated for 96 h at 27 °C and 120 rpm. After being harvested, supernatants were used to perform the penicillin bioassay, with *Staphylococcus aureus* as the indicator organism. The obtained mycelia were used to measure the dry weight. All experiments were performed in triplicate from at least two independent isolates.

## Results

### High-producer P2niaD18 has a duplicated penicillin biosynthesis cluster

**Fig. 1:**
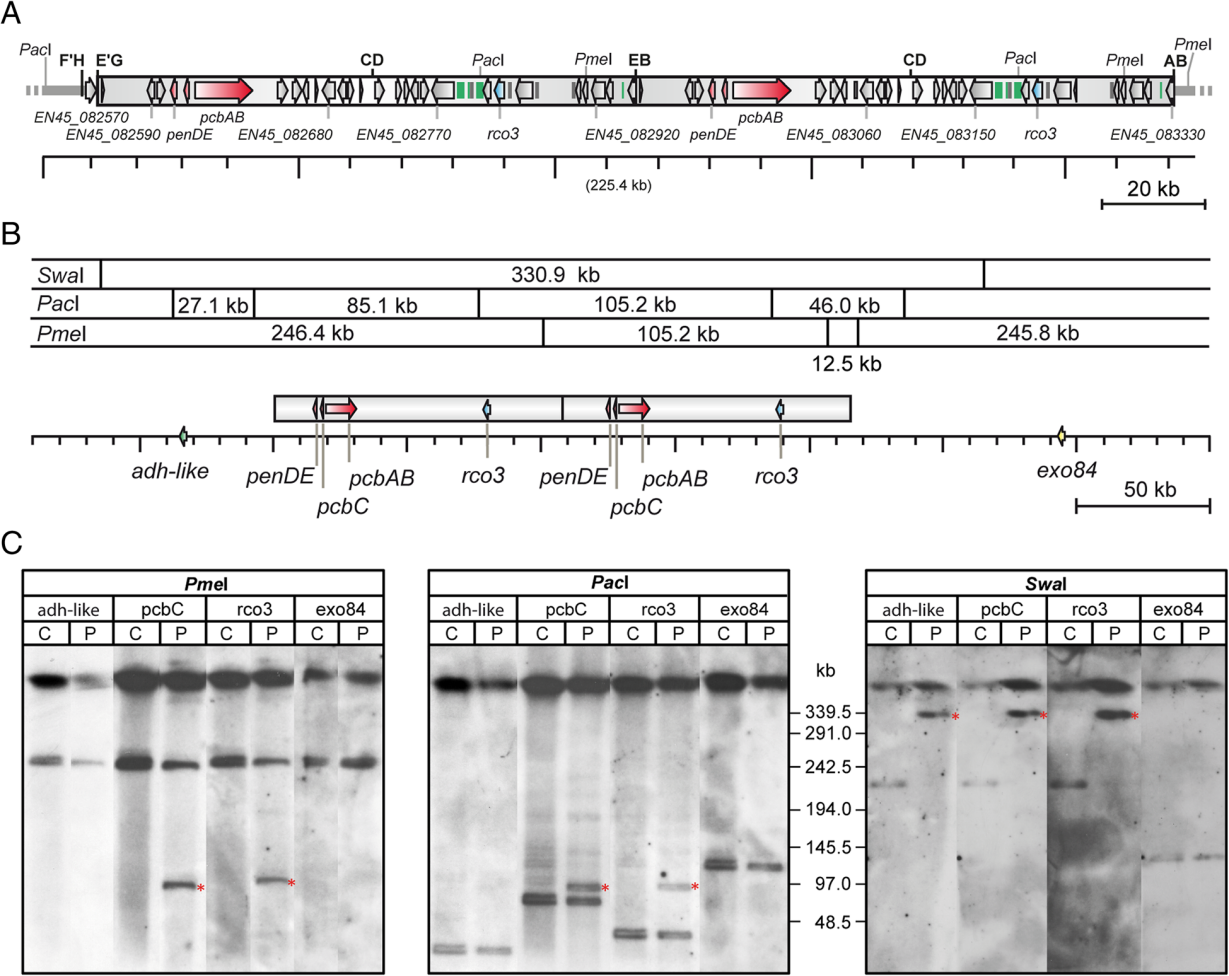
Determination of the copy-number of different *P. chrysogenum* strains using PFGE. (A) Genetic map of the duplicated 110 kb pen cluster region, based on the recently published genome sequence [23]. The penicillin biosynthesis genes are marked in red and recombination sites are indicated in bold face (**A-H**) and are identical to those reported for other Penicillium strains [9]. (B) Simplified map of the penicillin genes and adjacent regions showing the probes (*adh-like*, *pcbC*, *rco3* and *exo84*) for Southern hybridization experiments. In addition restriction sites for *Pme*I, *Pac*I and *Swa*I are given. (C) Pulsed field gel electrophoresis (PFGE) of *Pme*I, *Pac*I or *Swa*I restricted genomic DNA. For these experiments, the original cantaloupe strain NRRL 1951 [C] and the high-producer P2niaD18 [P] were used. The duplicated area is given as a *grey bar* in AB, and fragments that indicate the proposed duplications are marked with a *red asterisk* in C

Fungi have relatively small genomes on the order of about 30–40 Mb, and can be separated on a single gel by PFGE. This analysis can be extended by using rare cutting restriction enzymes, thus allowing determination of the chromosomal structure of a region of interest [29]. This approach is in particular reasonable for *P. chrysogenum*, which has only four chromosomes, because the two larger chromosomes with a size above 10 Mb are difficult to separate electrophoretically [30]. Here, we performed PFGE (Fig. 1A-C) to compare the copy number of the penicillin biosynthesis cluster in the high producer P2niaD18 with the wild-type strain NRRL 1951, the progenitor of all industrially used penicillin producers. P2niaD18 is a derivatives of P2, a former producer strain of Nippon Kayaku Co. Laboratories, and among the original strains of the Panlabs series [12]. P2 and Wisconsin 54-1255 are two independently derived derivatives from the very early penicillin production strain Q176, which was later used for further strain improvement programs [10, 12].

PFGE was conducted with chromosomal DNA of both strains cut with the three different rare cutting enzymes *Swa*I, *Pac*I, and *Pme*I. After PFGE, Southern analysis with four different probes (*adh-like* (Pc21g21650), *pcbC* (Pc21g21380), *rco3* (Pc21g21590), and *exo84* (Pc21g21980)) revealed several differences (Fig. 1B, C). For example, after restriction with both *Pme*I and *Pac*I, an additional signal with a size of about 97 kb was present in the genomic DNA of P2niaD18 after hybridization with the probes *pcbC* and *rco3*. After restriction with *Swa*I, a shift in the fragment size from about 230 kb to 340 kb occurred in P2niaD18 for all probes except *exo84* (Fig. 1C). These data confirmed that a genomic region of about 110 kb, which harbors the pen cluster, is duplicated in the high-producer strain compared to the wild-type strain. The recent genome sequence of P2niaD18 revealed that the two copies of the pen cluster are identical at the nucleotide level, and the corresponding gene map is given in Fig. 1A.

### Generation of a *pcbC* null mutant and different complementation strains

**Fig. 2:**
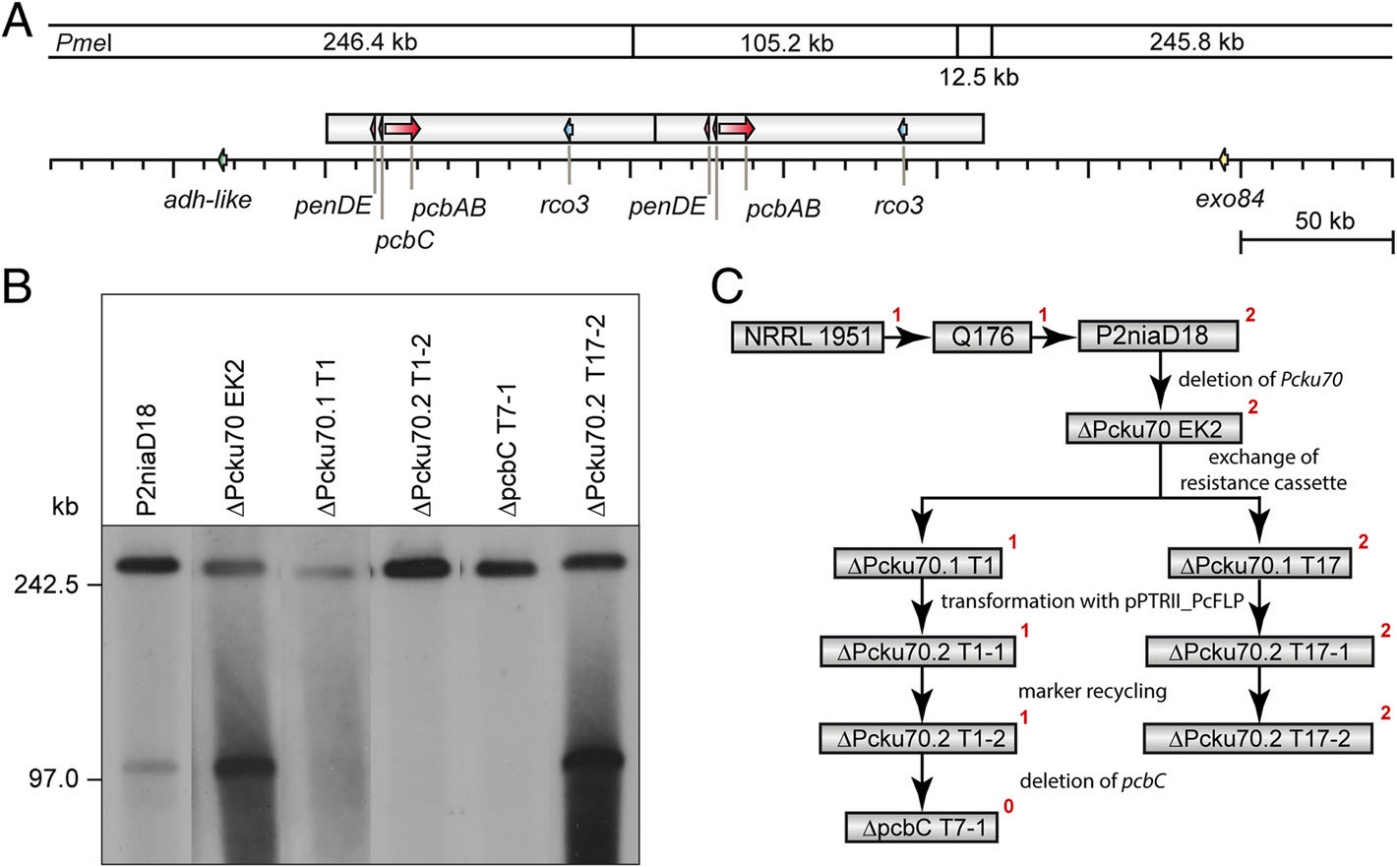
PFGE of P2niaD18 and its derivatives indicate loss of one cluster copy. **a** *Pme*I restriction map of the duplicated penicillin cluster. **b** PFGE was performed using restriction enzyme *Pme*I and a genomic fragment comprising the *pcbC* gene as a probe. For P2niaD18, this results in two signals with the size of 246.2 kb and 97 kb (**a**, **b**), thus indicating the duplication as proposed in **a**. During the generation of a marker-free ΔPcku70 strain, the extra copy of the penicillin cluster was lost in T1 and its derivatives. In contrast, T17 and its derivatives still carry both copies (**c**). In ΔpcbC, all copies of the *pcbC* gene were deleted, however a signal still occurs since the probe comprises the gene, the adjacent promotor region, and part of the flanking *penDE* gene. **c** Genealogy of penicillin production strains used in this study. The copy number of the penicillin gene cluster is given in *red*

In the next set of experiments, we constructed strains lacking the *pcbC* gene (Fig. 2a-c). ΔPcku70 EK2 served as parental strain for a series of derivatives, which are displayed in Fig. 2c and are described further in the following section. Previously, strain P2niaD18 was used to generate a ΔPcku70 strain for optimized homologous recombination, which was designated ΔPcku70 EK2 [31]. Both strains have a penicillin V titer of 3 g/L, when grown for 96 h in liquid shaking cultures [28]. ΔPcku70 EK2 was transformed with a flipper cassette to generate a marker-free ΔPcku70 strain [16] in two steps. First, the nourseothricin resistance cassette of ∆Pcku70 EK2 was replaced by a phleomycin resistance cassette flanked by *FRT* sites, resulting in two independent isolates, named ∆Pcku70.1 T1 and ∆Pcku70.1 T17 (Fig. 2c). Subsequently, both isolates were transformed with the free replicating plasmid pPTRII_PcFLP, which carries the *Pcflp* gene coding for the FLP recombinase, and isolates were named ∆Pcku70.2 T1-1 and ∆Pcku70.2 T17-1. Induction of the flipper-recombinase gene resulted in an excision of the phleomycin resistance cassette and the following loss of the free replicating plasmid results in the marker-free strains ∆Pcku70.2 T1-2 and ∆Pcku70.2 T17-2 [16]. PFGE revealed, after restriction with *Pme*I and subsequent hybridization with probe *pcbC* (comprising not only the *pcbC* ORF but also the promoter region and part of the *penDE* gene), that the primary transformant ∆Pcku70.1 T1, as well ∆Pcku70.2 T1-2 had lost one copy of the pen cluster (Fig. 2a, b). In contrast, ∆Pcku70.2 T17-2 still carries both copies. In the next step, we used ∆Pcku70.2 T1-2 to construct a marker-free *pcbC* null mutant. This strain, designated ΔpcbC T7-1, was obtained by homologous recombination using the flipper cassette (Additional file 1: Figure S1A).

The deletion of ΔpcbC was further confirmed by PCR (Additional file 1: Figure S1B) and Southern hybridization using the 3’ flanking region of *pcbC* as a probe (Additional file 1: Figure S1C). As an example, three independently derived transformants (T7, T9, and T10) are shown.

To generate penicillin-producing strains from the above-described null mutant, the *pcbC* gene under control of its native promoter was used for homologous recombination (Additional file 2: Figure S2A). The approach generated ΔpcbC::pcbC, of which five individual transformants (T1-T5) were tested. The site-specific integration of the *pcbC* genes was verified by PCR (Additional file 2: Figure S2B) and Southern hybridization (Additional file 2: Figure S2C). Additionally, we generated a penicillin-producing strain (ΔpcbC::pcbC-gfp) by ectopic integration of a *pcbC*-*egfp* fusion construct under control of the constitutive *gpd* promoter. Southern hybridization revealed at least three ectopic integrations of this construct (Additional file 2: Figure S2C, right lane).

**Fig. 3:**
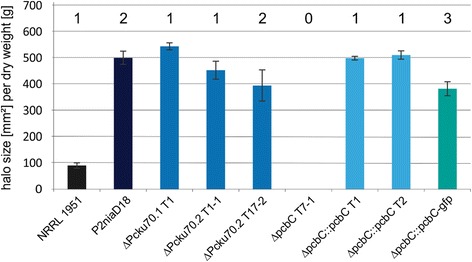
Quantification of penicillin production of strains with different copy numbers of the *pcbC* gene. The strains were grown for 96 h in shaking cultures. The diameter of each halo was measured to calculate the area and is given in relation to the dry weight of the respective culture. Standard deviations were determined from representative isolates, which were measured in triplicate. The copy number of each strain is displayed above the corresponding column. For all strains with a copy number of 1 or 2, the number indicates both the copy number of the *pcbC* gene and the complete cluster. For ΔpcbC T7-1, one copy of both *pcbAB* and *penDE* is still present, whereas *pcbC* is missing. To generate ΔpcbC::pcbC-gfp, the ΔpcbC recipient was complemented with three ectopic integrations of a *pcbC*-*egfp* fusion construct. For comparison, wild type strain NRRL 1951, which carries one copy of the penicillin cluster, is given

Our next step was to determine whether the copy number in these strains affected penicillin biosynthesis, so we conducted a halo assay using the indicator bacterium *Staphylococcus aureus*. The size of the halo was measured in relation to the dry weight of the mycelium. Most strikingly, no differences were detectable between the reference strain P2niaD18 and the strain ΔPcku70.2 T17-2, which both harbored two copies of the cluster, and two strains T1 (ΔPcku70.1) and T1-1 (ΔPcku70.2), which lack one copy (Fig. 3). As expected, disruption of the *pcbC* gene in ∆Pcku70.2 T1-2 yielded a penicillin non-producer (ΔpcbC T7-1), verifying that this strain is indeed single copy with respect to the penicillin biosynthesis gene cluster. Complementation of the null mutant with one copy of the *pcbC* gene under its native promoter resulted in penicillin biosynthesis comparable to the reference strains. Additionally, an ectopic integration of at least three copies of a *pcbC*-*egfp* fusion construct under control of the constitutive *gpd* promoter (ΔpcbC::pcbC-gfp) complemented the penicillin defect of the null mutant to an extent similar to that of the native complementation constructs (Fig. 3). For comparison, the titer of the wild type strain NRRL 1951 is given, which has a titer of about 20% in our plate assays compared with the other penicillin producing strains.

**Fig. 4:**
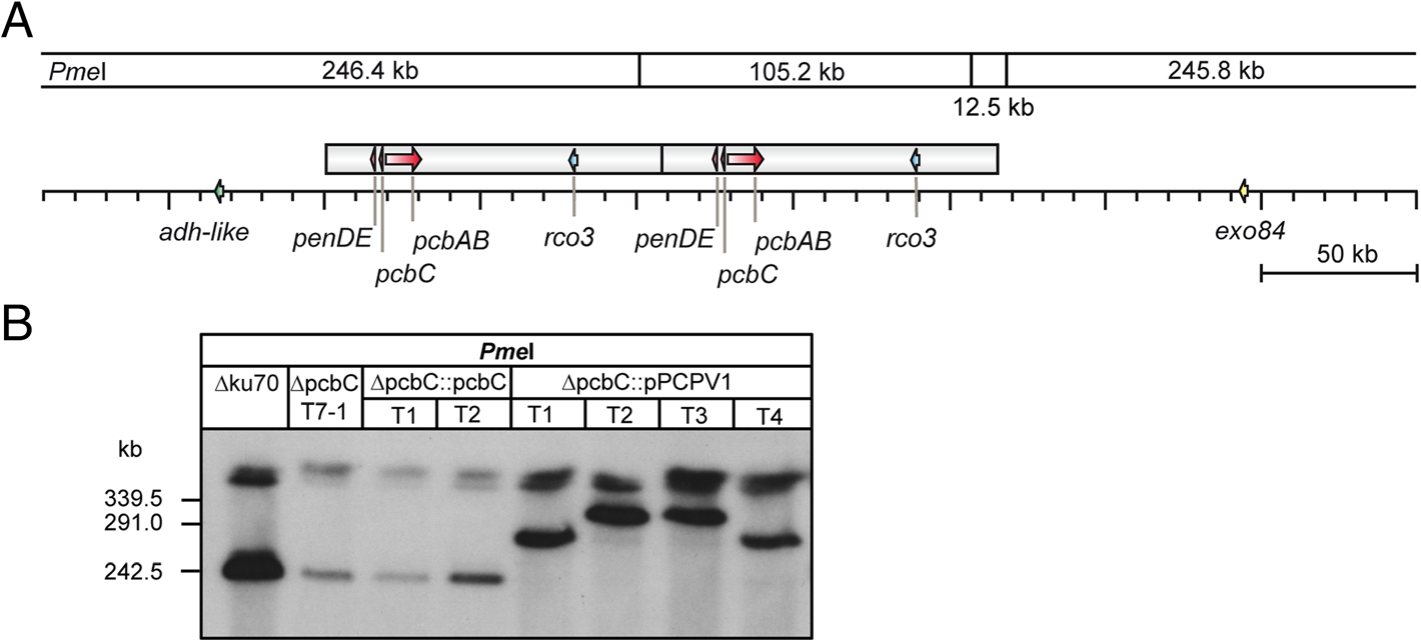
Generation of *pcbC* complementation strains by complementation with the complete penicillin biosynthesis cluster. The *pcbC* null mutant was complemented with plasmid pPCPV1 harboring the complete biosynthesis cluster (**a**). PFGE proved that integration of the plasmid occurred in the genomic region of the penicillin cluster and further showed single (T1, T4) or double (T2, T3) integration of the plasmid (**b**). The gene content of individual strains is indicated above each lane, for comparison strain Δku70 (ΔPcku70 EK2) is given

**Fig. 5:**
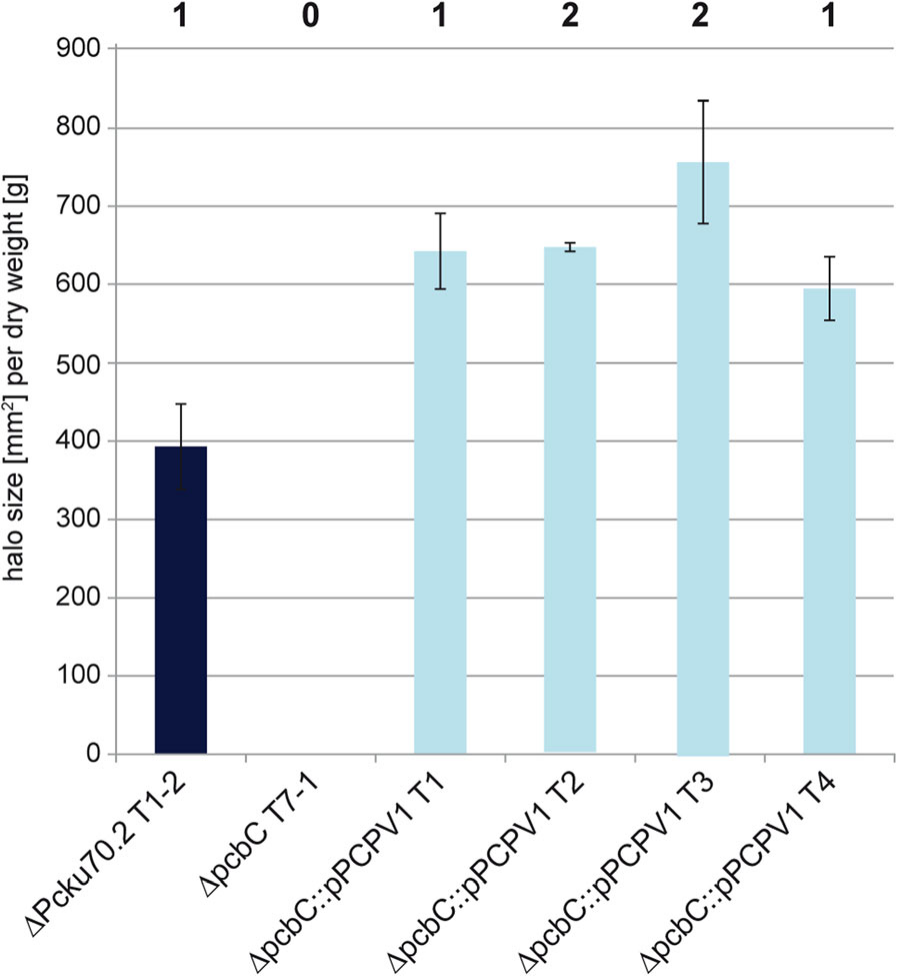
Quantification of penicillin production of complementation strains with different copy numbers of the penicillin biosynthesis cluster. Standard deviations were determined from representative isolates, which were measured in triplicate. The copy number of each strain is displayed above the corresponding column

We then complemented the ΔpcbC mutant with plasmid pPCPV1, which carries the complete penicillin biosynthesis cluster of 26 kb, together with the *niaD* gene, thus allowing complementation of the nitrate reductase deficiency of P2niaD18 and its descendants. After restriction of the chromosomal DNA with *Pme*I, followed by PFGE, the subsequent Southern hybridization with a probe comprising *pcbC* revealed that this construct integrated in all cases in the genomic area that harbors the native cluster. However, some transformants (T1, T4) showed a single integration of the plasmid whereas T2 and T3 carried two copies of the plasmid (Fig. 4), thus resulting in complementation strains with either one or two intact copies of the cluster. A penicillin bioassay again revealed no significant differences between the complementation strains, independent from the copy number (Fig. 5). All four complementation strains even slightly exceeded the penicillin production observed in the reference strain ΔPcku70.2 T1-2.

### Quantification of the *pcbC* transcript in deletion and complementation strains, carrying different copies of the penicillin cluster

**Fig. 6:**
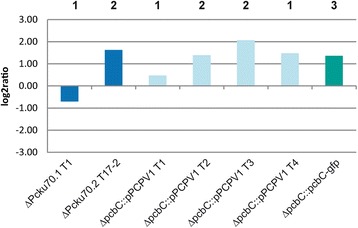
qRT-PCR analysis to quantify the *pcbC* transcript. RNAs, isolated from strains as indicated, were used for quantification. The procedure is described in the Material and Methods section, with oligonucleotides as listed in Table 3. SSUrRNA served as reference for normalization. The copy number of each strain is displayed above the corresponding column

Finally, we tested whether the transcriptional expression level reflects the penicillin titer observed in the recipient and recombinant strains. All strains were grown for 3 days in shaking flasks with rich Complete Culture Medium (CCM). This time point represents the early phase of β-lactam production and is characterized by an expressional switch from genes related to vegetative growth to those involved in secondary metabolite formation [17]. As shown in Fig. 6, we quantified the *pcbC* transcript from three biological replicates in relation to the SSUrRNA. Both pcbC null mutants (ΔpcbC T7.1 + T10) gave negative results, and the single copy strain ΔPcku70.1 T1 showed an almost zero expression level. The results of the quantitative PCR analysis correspond roughly to the copy number of the *pcbC* gene. The four independently derived transformants ΔpcbC::pPCPV1 T1-T4 have similar transcript levels although distinct by the copy number of their penicillin gene cluster. This analysis of relative log2-fold expression ratios of the *pcbC* transcript support our previous results that copy number and thus transcript level have only a minor effect on penicillin titers.

## Discussion

The three penicillin biosynthesis genes *pcbAB*, *pcbC*, and *penDE* are clustered in a single 18-kb region in wild-type strains of the filamentous fungi *P. chrysogenum* and *Aspergillus nidulans*. Previous chromosome separation and DNA hybridization analysis showed that production strains from *P. chrysogenum* have up to 14 copies of a 56.8-kb region carrying further protein coding genes that are not characterized in detail [15, 32]. This high copy number was suggested to be relevant for the high penicillin titer observed [11]. Our analysis, however, indicates that regulatory genes unrelated to the penicillin biosynthesis gene cluster are responsible for increased penicillin production, at least in the P2 line of production strains.

Theilgaard et al. [33] showed that penicillin production in the low-producing, single gene copy strain Wisconsin 54-1255 could be increased by integration of additional copies of the three penicillin biosynthesis genes. However, for increased titer, all three genes had to be integrated; other combinations with only one or two of the genes did not result in higher penicillin production. The authors proposed that amplification of all three biosynthesis genes is responsible for the high penicillin titer of production strains. More recently, it was found that the amount of penicillin V increases with penicillin biosynthetic gene cluster number but with saturation at high copy numbers. This study was done in industrial strains with a Wisconsin 54-1255 background [34]. Remarkably, in that study, the protein level of the acyltransferase, the gene product of *penDE*, was saturated already at low cluster copy numbers, suggesting that the acyltransferase reaction presents a bottleneck in the biosynthesis process.

Several high-production strains have been described that comprise multiple copies of the penicillin cluster [3]. For example, strain AS-P-78 carries between five and nine copies [11, 15]. Another strain, BW 1890, harbors between 8 and 16 copies of the cluster [32]. The Panlabs strain P2, which is also a derivative of Q176, was thought to carry between 5 and 14 copies [11, 15, 22]. However, genome analysis and results from our work revealed that at least strain P2niaD18, a nitrate reductase–deficient derivative that emerged from P2 by conventional mutagenesis, harbors only two copies of the penicillin biosynthesis cluster [14]. Still, this strain produces high amounts of penicillin, indicating that copy number is not the sole factor in increased production rates. Our data strongly support this conclusion, revealing that even the loss of one of the two copies did not result in significantly decreased penicillin biosynthesis. In addition, complementation strains with one or two copies of the cluster yielded no significant differences in titer.

The amplified regions might even be responsible for the genetic instability of strains with multiple copies of the biosynthetic gene clusters. Harris et al. [35] described, for example, that after protoplasting, gene clusters are easily lost in industrial strains derived from Wisconsin54-1255. Similar observations were made for the yeast *Yarrowia lipolytica*. To test gene amplification in the rDNA of *Y. lipolytica*, several plasmids were transformed into the yeast cells [36]. Among other elements, these plasmids harbored the reporter gene *XPR2* encoding alkaline extracellular protease (AEP). Plasmid copy number was stable for strains containing fewer than 10 copies per cell. However, for higher copy numbers, multiple integrations were stable only when AEP synthesis was not induced, while in inducing medium, the stability of the multiple integrations was dramatically affected. After AEP induction, a reduced growth rate was observed, suggesting that the increased secretory pathway cargo load influenced cell growth.

These data together with our observation that one of two penicillin biosynthesis clusters was randomly lost support the hypothesis that the cluster copies are easily lost and that high-copy strains are unstable. P2niaD18 is a strain with only two copies but nevertheless capable of high penicillin production, indicating that other factors have an important influence on penicillin biosynthesis.

To date, several regulators of penicillin biosynthesis are already known in filamentous fungi ([2], reviewed in [5]). So far, a pathway specific regulator of the penicillin biosynthesis cluster has not yet been described. In addition to positively acting global regulators like the pH-dependent transcriptional activator PACC and the CCAAT binding complex AnCF [37–40], proteins of the velvet family have become of special interest as repressors in recent years [41]. These regulatory proteins play a key role in coordinating secondary metabolism and differentiation processes such as sexual and asexual sporulation in various filamentous fungi (reviewed in [42]). In *Aspergillus nidulans*, VeA forms a heterotrimeric complex with VelB, another protein of the velvet family, and the global regulator LaeA under dark conditions to control sexual development and secondary metabolism [43, 44]. In addition, VelB interacts with VosA, a third velvet-like protein, to form a subcomplex that is essential for asexual and sexual spore formation as well as trehalose biogenesis [44, 45]. Meanwhile, homologs of velvet components have been identified in numerous other filamentous fungi (for an overview see [42]. In *P. chrysogenum*, the velvet proteins control hyphal morphogenesis, conidiophore development, and penicillin biosynthesis. Most importantly, distinct velvet proteins either activate or repress biosynthesis of penicillin [21, 46]. Interestingly, in another industrial fungus, the ascomycete *Acremonium chrysogenum*, a velvet homologue has a regulatory role on beta-lactam antibiotic production [47]. In this fungus, at least seven genes for the biosynthesis of the beta-lactam antibiotic cephalosporin C are located on two different clusters on different chromosomes [23]. Thus, simple amplification of a single cluster will not increase cephalosporin C biosynthesis. Molecular analysis by different investigators has already shown that global regulators are responsible for high titer of cephalosporin C biosynthesis (for review see [48]). Recently, we found a rather unexpected regulation of gene expression. The mating type locus encoded MAT1-1-1 transcription factor is known for its role in sexual identity. However, recent investigations showed a transcriptional control of wide range of genes with biotechnological relevance including those regulating penicillin production. Compared with control strains, mutants lacking the mating type locus showed a significant reduction in penicillin biosynthesis throughout a time course [28, 49].

## Conclusions

This report reveals that a high copy number of the three structural genes and an increased *pcbC* transcript level are not strict prerequisites for increased penicillin production in the production strain *Penicillium chrysogenum* P2niaD18. Most strikingly, a loss of one of the two identical copies of the cluster did not significantly influence the amount of penicillin produced. These data imply that copy number is not the limiting factor for increased penicillin biosynthesis in the strains investigated and we anticipate that instead, wide domain regulatory factors *in trans* are involved in this process and are thus important targets for future strain improvement.

## Additional files

Additional file 1:
**Figure S1.** Construction of *pcbC* null mutant. (TIF 953 kb)
Additional file 2:
**Figure S2.** Construction of *pcbC* single- and multi-copy strains, using ΔpcbC. (TIF 1242 kb)

## Abbreviations

ACV: δ-(L-α-aminoadipyl)-L-cysteinyl-D-valine
AEP: Alkaline extracellular protease
ATCC: American Type Culture Collection
CBS: Centraalbureau voor Schimmelcultures
CCM: Complete culture medium
FRT: FLP recognition target
NRRL: ARS culture collection
ORF: Open reading frame
PCR: Polymerase chain reaction
PFGE: Pulsed-field gel electrophoresis
qRT-PCR: quantitative reverse transcription PCR
SSUrRNA: Small subunit ribosomal RNA
UV: Ultraviolet
