# Supplementary figures and images for: Penicillin production in industrial strain Penicillium chrysogenum P2niaD18 is not dependent on the copy number of biosynthesis genes
Source: BMC Biotechnol. 2017 Feb 16;17:16. doi: 10.1186/s12896-017-0335-8 (PMC5314624; doi:10.1186/s12896-017-0335-8)

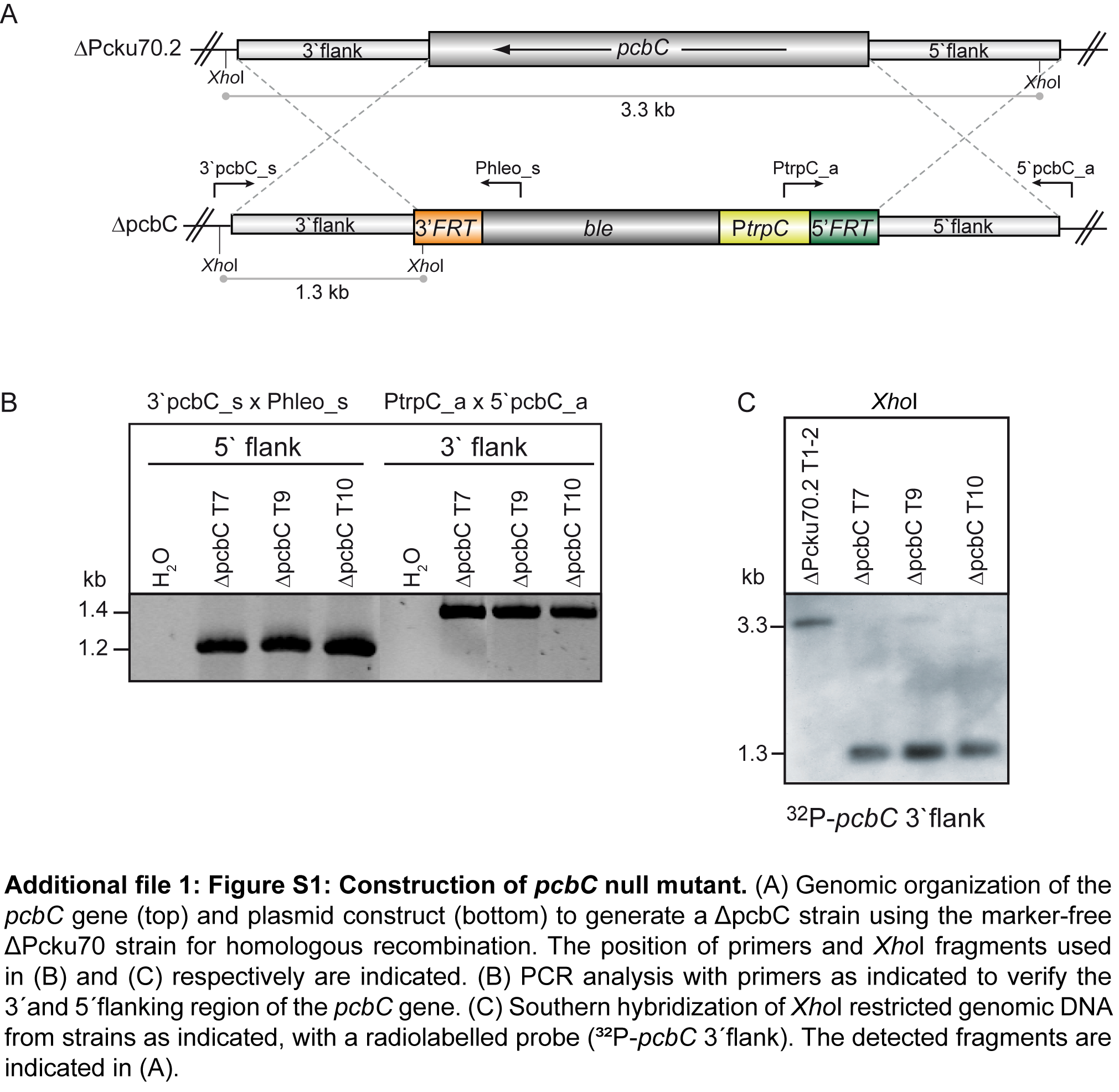

Supplement: Additional file 1: — Figure S1. Construction of pcbC null mutant. (TIF 953 kb) [file 12896_2017_335_MOESM1_ESM.tif]

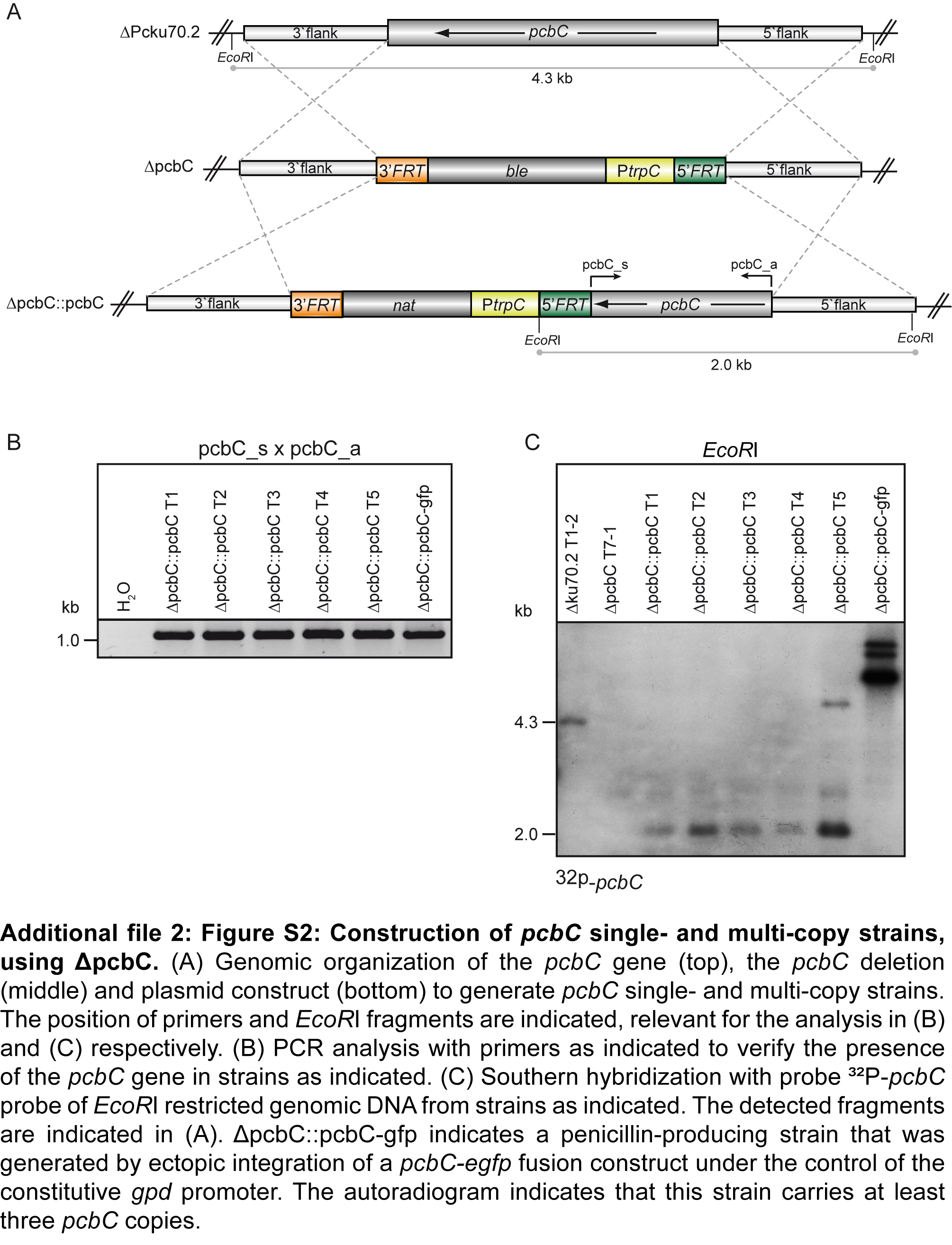

Supplement: Additional file 2: — Figure S2. Construction of pcbC single- and multi-copy strains, using ΔpcbC. (TIF 1242 kb) [file 12896_2017_335_MOESM2_ESM.tif]
